# Mixed drug overdose involving clonazepam, alprazolam, and olanzapine in a 72-year-old male with Parkinson’s disease: a case report

**DOI:** 10.3389/fnins.2025.1570726

**Published:** 2025-06-04

**Authors:** Wei Zhang, Mao-Shui Wang, Jianguo Zhang, Chunhai Gao

**Affiliations:** ^1^Orthopaedic Medicine Center, Linyi People’s Hospital, Shandong Second Medical University, Linyi, China; ^2^Department of Lab Medicine, Shandong Public Health Clinical Center, Shandong University, Jinan, China; ^3^Department of Critical Care Medicine, Linyi People's Hospital, Shandong Second Medical University, Linyi, China; ^4^Department of Laboratory Medicine, Key Laboratory for Laboratory Medicine of Linyi City, Shandong Provincial Medicine and Health Key Laboratory for Precise Diagnosis of Hereditary Rare Diseases, Linyi People’s Hospital, Shandong Second Medical University, Linyi, China

**Keywords:** elderly patient, clonazepam, overdose, renal replacement therapy, pneumonia

## Abstract

This case report discusses a 72-year-old male patient with Parkinson’s disease, depression, and somatization disorder, presenting to the emergency department after a suspected overdose of clonazepam. The patient was discovered unconscious approximately 2.5 h after ingestion of over 30 tablets of clonazepam, in combination with an overdose of alprazolam and olanzapine. Despite initial challenges with gastric lavage, subsequent treatment involved supportive care, management of complications such as pneumonia, and renal replacement therapy, ultimately leading to recovery. This case highlights the complexities of managing overdoses, particularly those involving drugs with long half-lives, and highlights the challenges of treating elderly patients and the importance of supportive care in recovery.

## Introduction

Benzodiazepines, such as clonazepam, are frequently prescribed for conditions like anxiety, insomnia, and seizure disorders. However, overdose can result in life-threatening central nervous system depression (Edinoff et al., 2021). The elderly population is particularly at risk due to age-related changes in drug metabolism and the presence of multiple comorbidities (Airagnes et al., 2016; Singh and Sarkar, 2016).

Psychotic disorders are associated with serious outcomes, including suicide, homicide, and other high-risk behaviors. In a Swedish cohort study, benzodiazepine use was predominant among individuals with psychotic disorders who died by suicide (Carlsten et al., 2003). Due to its high potency and intermediate to long duration of action, clonazepam has become one of the most widely used treatments for anxiety-related disorders and seizures (Mehdi, 2012; Perna et al., 2016).

This report presents a case with overdose of clonazepam. This study highlighted the management of a severe clonazepam overdose in a 72-year-old male with Parkinson’s disease, highlighting the challenges posed by advanced age, comorbid conditions, and polypharmacy. The study is a retrospective observational case report. No therapeutic interventions were administered as part of the study protocol. Standard supportive care was provided based on clinical judgment; no study-specific interventions were undertaken.

## Case presentation

On November 7th, an elderly male patient (71 years old) was admitted to our emergency department in a comatose state. The patient had a history of Parkinson’s disease, depression, and somatization disorder. Prior to admission, his regular medications comprised idebenone (30 mg, three times daily, orally), escitalopram oxalate (10 mg, once daily, orally), trazodone (50 mg, nightly, orally), levodopa/carbidopa sustained-release tablets (250 mg, nightly, orally), levodopa/benserazide (125 mg, three times daily, orally), pramipexole (0.5 mg, three times daily, orally), and clonazepam (1 mg, nightly, orally). He had no history of hypertension, diabetes, smoking, or alcohol consumption. According to his family, the patient was suspected to have ingested over 30 tablets (1 mg) of clonazepam, and they found him unconscious approximately 2.5 h later.

The patient’s regular medications included idebenone, escitalopram, trazodone, carbidopa-levodopa, aspirin, atorvastatin, pramipexole, and clonazepam. Recently, he had shown signs of emotional instability, guilt, and self-blame, raising concerns of depressive exacerbation.

### Initial management

Upon arrival, gastric lavage was attempted twice but was unsuccessful due to both attempts entering the respiratory tract, leading to the decision to abandon the procedure. Laboratory evaluations showed no abnormalities in blood counts, C-reactive protein (CRP), coagulation profile, electrolytes, liver and renal function tests. A brain MRI (Supplementary Figure 1) revealed no abnormalities, and the patient’s vital signs were stable: body temperature 36.5°C, respiratory rate 18 breaths per minute, heart rate 72 beats per minute, blood pressure 110/73 mmHg, and oxygen saturation of 99%.

Due to the refusal of renal replacement therapy by the family, intravenous fluid administration was initiated to accelerate drug elimination, and Flumazenil was administrated with 0.01 mg/kg. The next day, on November 8th, the patient developed a fever of 39.5°C. Laboratory findings showed elevated white blood cell counts 10.92 (ref, 3.5–9.5) *10^9^/L, CRP 13.2 (ref, 0–8) mg/L, and positive influenza B IgM, as well as lung inflammation evident on chest CT scans (Supplementary Figure 2). Additionally, elevated clonazepam (121 ng/mL; ref. 0–20), alprazolam (135 ng/mL; ref. 0–20), and olanzapine (758 ng/mL, ref. 0–100) levels were noted, which confirmed the overuse of clonazepam.

The patient was then transferred to the ICU for further management. The following day, coagulation abnormalities were noted: prothrombin time was 20.30 s (ref, 11.8–15.1 s), APTT was 45.60 s (ref, 28–42.5 s), fibrinogen was 4.39 g/L (ref, 2–4 g/L), D-dimer was 2.47 μg/mL (ref, 0–0.5 μg/mL), and fibrin degradation products were 9.56 μg/mL (ref, 0–5.0 μg/mL). Additionally, a sputum culture revealed an *Acinetobacter baumannii* infection, with drug susceptibility testing indicating sensitivity to tigecycline and minocycline. The patient was started on omadacycline (loading dose: 200 mg; maintenance dose: 100 mg), and renal replacement therapy.

### Clinical course

On November 10th, due to decreasing oxygen saturation (88%), the patient was intubated and started on mechanical ventilation. Following four rounds of renal replacement therapy, the patient gradually regained consciousness, and on November 12th, he was extubated and transitioned to high-flow oxygen therapy and administrated with carbidopa-levodopa for Parkinson’s disease.

On November 13th, the patient was transferred to the respiratory department for continued antibiotic therapy (omadacycline) and subsequently discharged in stable condition and prescribed an additional course of minocycline (minocycline: loading dose: 200 mg; maintenance dose: 100 mg/12 h for 7 days).

## Discussion

This case highlights the complexity of managing clonazepam overdose in elderly patients, particularly those with multiple comorbidities such as Parkinson’s disease and pneumonia. Clonazepam exhibits rapid absorption following oral administration, with peak plasma concentrations typically achieved within one to four hours post-dose. The elimination half-life of clonazepam ranges from 30 to 40 h (ResearchGate, 2025.). The prolonged metabolism of clonazepam in elderly patients contributes to extended recovery times, as evidenced by the patient taking six days to regain full consciousness. According to the literature, benzodiazepines such as clonazepam and other CNS depressants have longer half-lives in elderly individuals due to decreased hepatic clearance and renal function, which significantly prolongs drug elimination and potentiates toxicity (Bourin, 2021). Similarly, it was reported that inappropriate dosage adjustments and drug abuse in elderly patients can lead to severe outcomes, which necessitates close monitoring and individualized intervention strategies (Li et al., 2022). In addition, the increasing prevalence of illicit benzodiazepine use is a growing public health concern, contributing to higher rates of overdose. Benzodiazepine-involved emergency department (ED) visits per 100,000 ED visits increased by 23.7% from 2019 to 2020, and associated mortality also rose (Liu et al., 2021). Overall benzodiazepine-related deaths increased by 42.9%, from 1,004 to 1,435, between April–June 2019 and April–June 2020 (Liu et al., 2021). Benzodiazepines, when obtained outside of prescribed medical contexts, often lead to dangerous combinations with other substances, significantly increasing the risk of respiratory depression, coma, and death (Brandt and Leong, 2017). This highlights the need for improved awareness, preventive measures, and public health strategies to combat the misuse of benzodiazepines and other central nervous system depressants.

One major complication encountered during this patient’s management was pneumonia, which likely developed due to prolonged bed rest and impaired pulmonary function (Chughtai et al., 2017; Jang et al., 2019). This highlights the importance of early mobilization, sputum clearance, and antibiotic prophylaxis in patients who are at high risk of respiratory complications. Effective management of pneumonia and other potential infections is essential in improving patient outcomes in cases of prolonged unconsciousness or mechanical ventilation (Battaglini et al., 2021). Fortunately, not all of the drugs tested were ineffective against the isolated *Acinetobacter baumannii.*

Renal replacement therapy played a crucial role in this patient’s recovery, which could facilitate the clearance of clonazepam and other medications present in elevated concentrations, such as alprazolam and olanzapine. The use of renal replacement therapy helped to accelerate the reduction of toxic levels of these drugs, contributing to the patient’s eventual awakening. Early implementation of renal replacement therapy in similar cases may help reduce toxicity and improve recovery outcomes, as supported by Mirrakhimov et al. (2016), who demonstrated the efficacy of renal replacement therapy in managing combined intoxications involving multiple CNS depressants.

The patient’s age and comorbidities presented unique challenges in managing the overdose, and careful consideration of these factors was necessary to navigate the clinical course. The presence of Parkinson’s disease added complexity to his medication regimen, which included multiple drugs with potential interactions. This case emphasizes the importance of careful medication management and the risks associated with polypharmacy, especially in elderly patients with multiple health conditions. The report by Kobayashi et al. illustrates the dangers of polypharmacy in similar patient populations, showing how interactions among multiple CNS-active drugs can exacerbate symptoms and prolong recovery (Kobayashi et al., 2012). In addition, during ICU stay, Flumazenil was not administrated again due to risk of convulsions.

In general, despite the significant overdose, the patient maintained relatively stable vital signs throughout much of the hospitalization. Previously, concurrent use of olanzapine and clonazepam is known to elevate the risk of central nervous system and respiratory depression, particularly when administered simultaneously or without appropriate monitoring (Hayes, 2024; Yağci et al., 2017). Nevertheless, recent studies have reported that coadministration of olanzapine and clonazepam does not significantly increase the severity of adverse drug reactions (Liu et al., 2022; Yukang et al., 2021), which may be explained by the careful timing, dosing, and monitoring. Notably, advanced age and multiple comorbidities made it challenging to predict the clinical course accurately. Moreover, the decision to delay renal replacement therapy may have contributed to slower drug clearance and extended the time required for the patient to regain consciousness. This underscores the importance of timely and comprehensive supportive care in mitigating severe complications and improving patient outcomes.

## Conclusion

This case highlights the complexities associated with multiple drug overdoses and the challenges of managing benzodiazepine overdose in elderly patients with multiple comorbidities. It underscores the critical need for prompt, multidisciplinary intervention when managing such cases. Early supportive treatment, vigilant monitoring for potential complications, and the timely initiation of renal replacement therapy are essential for optimizing recovery outcomes. Further research is warranted to establish evidence-based protocols for the management of benzodiazepine overdose in similar high-risk populations.

## Data Availability

The original contributions presented in the study are included in the article/Supplementary material, further inquiries can be directed to the corresponding author.
