# Supplementary figures and images for: Mixed drug overdose involving clonazepam, alprazolam, and olanzapine in a 72-year-old male with Parkinson’s disease: a case report
Source: Front Neurosci. 2025 Jun 4;19:1570726. doi: 10.3389/fnins.2025.1570726 (PMC12174427; doi:10.3389/fnins.2025.1570726)

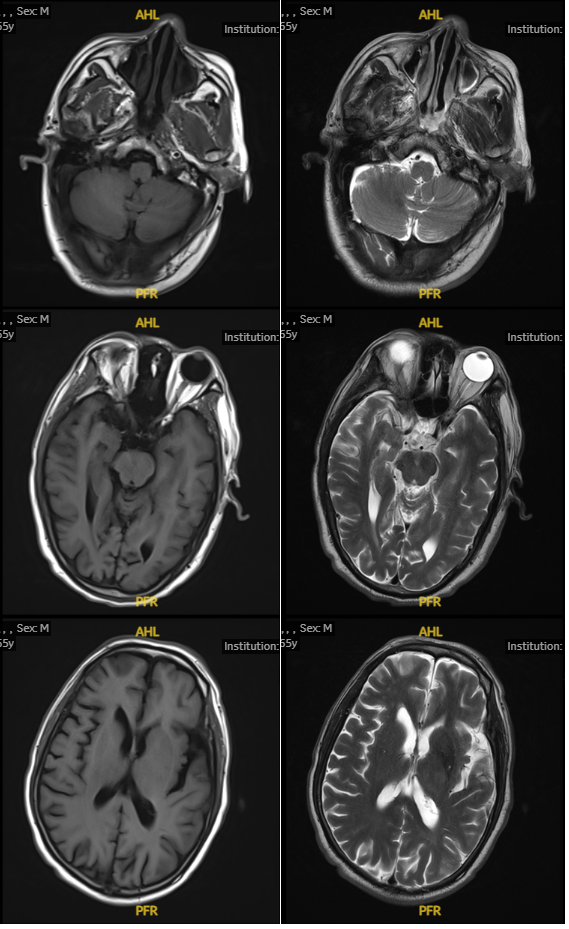

Supplement: Supplementary file 1 [file Image_1.png]

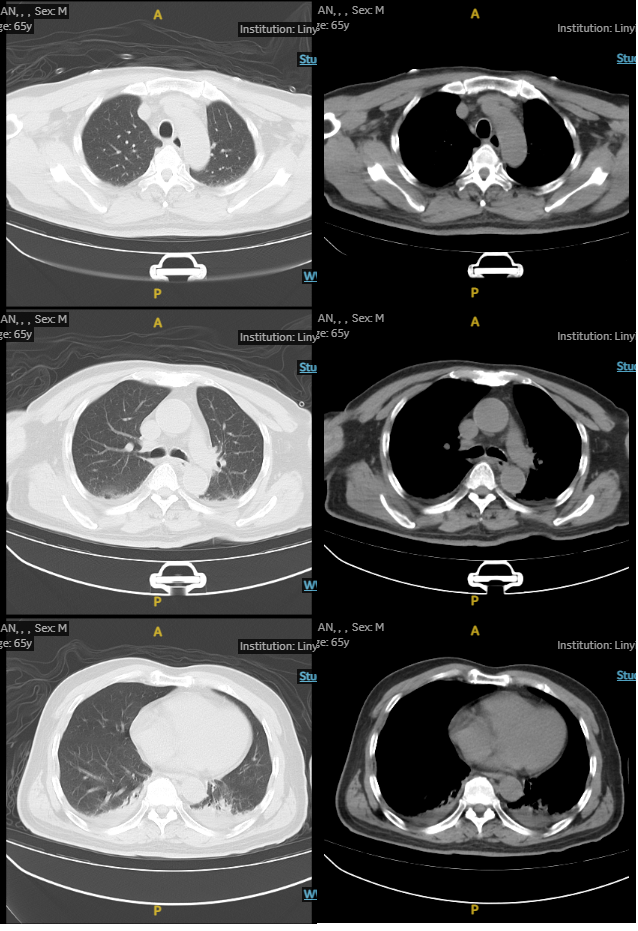

Supplement: Supplementary file 2 [file Image_2.png]
